# RNA Graph Partitioning for the Discovery of RNA Modularity: A Novel Application of Graph Partition Algorithm to Biology

**DOI:** 10.1371/journal.pone.0106074

**Published:** 2014-09-04

**Authors:** Namhee Kim, Zhe Zheng, Shereef Elmetwaly, Tamar Schlick

**Affiliations:** Department of Chemistry and Courant Institute of Mathematical Sciences, New York University, New York, New York, United States of America; Technical University Darmstadt, Germany

## Abstract

Graph representations have been widely used to analyze and design various economic, social, military, political, and biological networks. In systems biology, networks of cells and organs are useful for understanding disease and medical treatments and, in structural biology, structures of molecules can be described, including RNA structures. In our RNA-As-Graphs (RAG) framework, we represent RNA structures as tree graphs by translating unpaired regions into vertices and helices into edges. Here we explore the modularity of RNA structures by applying graph partitioning known in graph theory to divide an RNA graph into subgraphs. To our knowledge, this is the first application of graph partitioning to biology, and the results suggest a systematic approach for modular design in general. The graph partitioning algorithms utilize mathematical properties of the Laplacian eigenvector (µ2) corresponding to the second eigenvalues (λ2) associated with the topology matrix defining the graph: λ2 describes the overall topology, and the sum of µ2′s components is zero. The three types of algorithms, termed *median*, *sign*, and *gap* cuts, divide a graph by determining nodes of cut by median, zero, and largest gap of µ2′s components, respectively. We apply these algorithms to 45 graphs corresponding to all solved RNA structures up through 11 vertices (∼220 nucleotides). While we observe that the median cut divides a graph into two similar-sized subgraphs, the sign and gap cuts partition a graph into two topologically-distinct subgraphs. We find that the gap cut produces the best biologically-relevant partitioning for RNA because it divides RNAs at less stable connections while maintaining junctions intact. The iterative gap cuts suggest basic modules and assembly protocols to design large RNA structures. Our graph substructuring thus suggests a systematic approach to explore the modularity of biological networks. In our applications to RNA structures, subgraphs also suggest design strategies for novel RNA motifs.

## Introduction

Ribonucleotide Acid (RNA) has become a prominent subject in modern biology, due to recent discoveries of RNA’s vital roles in regulating gene expression, which come in addition to well-known roles in protein synthesis [1], [2], [3]. Based on these new discoveries, new applications are being pursued in areas such as therapeutic biotechnology, by using RNA’s editing, silencing, and other regulatory capabilities to activate and deactivate genes, deliver drugs, and design new nanomaterials [4], [5]. Like other molecules, all of these functions of RNA are closely tied to the three-dimensional structures that RNAs adopt. Thus, to explore these new potential functions of RNA, it is essential to understand the principles of RNA’s architecture. Such an understanding can naturally lead to RNA design as well, another area of intense current interest.

At the heart of RNA structure is its modularity [6], [7], [8]. RNA’s diverse structures are generated by the combination of recurrent modules on three different levels: sequence (1D), secondary (2D), and tertiary (3D) structures. RNA is a single-stranded polymer whose sugar-phosphate backbone with contains four primary building blocks, Adenine (A), Guanine (G), Uracil (U) and Cytosine (C). Modified bases also occur. This single-stranded polymer folds upon itself, to form GC, AU, or GU (“wobble”) base pairs which define double-helical regions (“stems”), imperfect with single-stranded regions named “hairpin loops”, “internal loops”, and “junctions”, with one, two, or more adjacent helical arms, respectively on the 2D (or base-pairing) level. Through other interactions in space, complex 3D structures form. Several 3D modules called motifs (e.g., coaxial helix, A-minor, ribose zipper, kissing hairpin, right-angles, twist-joint and double twist-joints motifs) have been identified by manual and computational inspection from experimentally resolved structures.

**Figure 1:**
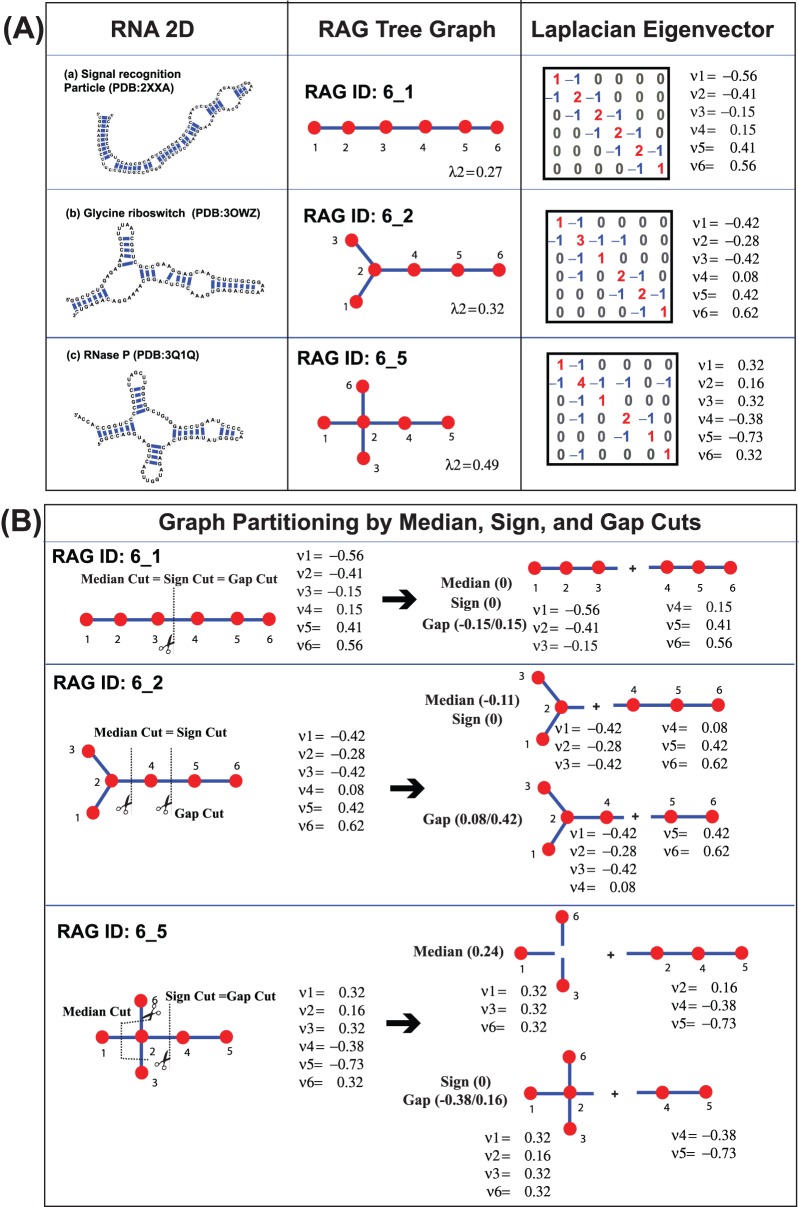
RNA-As-Graph (RAG) tree representation of RNA secondary structures and graph partitions using the second Laplacian eigenvector. (A) Examples of RNA secondary structures, their corresponding RAG tree graphs, Laplacian matrices, and the second eigenvectors: (a) signal recognition particle (PDB code: 2XXA, Graph ID: 6_1); (b) glycine riboswitch (PDB code: 3OWZ, Graph ID: 6_2); (c) RNase P (PDB code: 3Q1Q, Graph ID: 6_5). (B) Graph partitioning using the cut values of the median, sign, or the largest gap of the second Laplacian eigenvector with examples of three RAG graphs 6_1, 6_2, and 6_5.

Such modularity and hierarchy offers us a solid ground for conceptual and mathematical methods, such as graph theory, to investigate RNA’s structural repertoire. Graph theory is a well-established field of mathematics, which has been used extensively in a variety of economic, social, engineering, biological, and medical contexts to describe and analyze complex networks [9], [10], [11], [12]. Essentially, the foundations of graph theory can be used to enumerate and analyze combinatorial properties of networks [13], [14], [15], [16]. In the field of RNA structure, Waterman pioneered the development of the graphical representation of RNA primary/secondary network on the base level [17], and Shapiro and coworkers extended a tree representation of RNA secondary network at the base-pair level to measure structural similarity [18]. More recently, Schlick and coworkers developed the RNA-As-Graph (RAG) framework and web resource (http://www.biomath.nyu.edu/rna) to represent global RNA topologies as graphs (see Figure 1) [19], [20]. RAG has been pursued to enumerate, analyze, and predict RNA topologies, expanding our understanding of RNA’s structural repertoire. Interesting applications include prediction of RNA-like topologies [20], [21], [22], [23], prediction of non-coding RNA [24], [25], in silico modeling of the in vitro selection process for RNA design [26], [27], [28], analysis of large viral RNA [29], [30], and riboswitch analysis and design [31], [32] (see reviews [33], [34]). Recently, RAG 2D graph representations have been extended to 3D, and the substantial reduction in conformational space size has been exploited to enhance the sampling of 3D topologies to predict helical arrangements of RNA [35], [36].

In RAG, RNA structures are simplified as tree or dual graphs by translating RNA modules to graph theory objects such as vertices and edges. When helices are translated to edges and other modules are translated to vertices, RNA structures are represented as tree graphs (see Figure 1A). Using graph theory, all possible 2D topologies of RNAs can be enumerated [19], [20].

The Laplacian matrix of a graph provides a quantitative framework to describe the topology. In particular, the second eigenvalue of the Laplacian matrix has been used as a topological descriptor of RNA [20], [21]. The enumerated repertoire of tree-graph motifs has made possible classification of these motifs as existing (experimentally found) and hypothetical. Using the Laplacian eigenvalues as variables, we have used clustering analysis of RNA graphs to predict which of the remaining hypothetical motifs are “RNA-like” [19], [20], [21]. RNA-like graphs have been investigated to design targeted pools for in vitro selection [28] and have been merged to design larger RNA-like topologies [23].

Here, we present another application of graph theory to explore the modularity of RNA structures by partitioning RNA graphs using graph theory methods. The analysis of RNA structures at large has identified modular RNA structures, which are composed of repetitive motifs, in which patterns appear hierarchically from 2D to 3D structural levels [7]. Thus, a build-up of existing modules is a natural way to produce new structures by fragment assembly approaches, as we have done previously [21], [37]. However, so far, only a limited number of known motifs have been found by manual inspection, and there is no systematic way to divide whole RNAs into pieces for fragment assembly. Toward this goal, we apply graph partitioning methods to RNA tree graphs. Our computational approach divides large RNA structures into small recurrent motifs based on spectral graph partitioning.

The mathematical theory of graph partitioning is well developed [38], but the application of the graph partition algorithm to biology has not been attempted as far as we are aware. Because the topological properties of RNA graphs can be described by the second eigenvalue (λ2) of the Laplacian matrix, we utilize the eigenvector (µ2) of the Laplacian matrix corresponding to λ2. This eigenvector µ2 provides us information on how to divide a graph into smaller fragments that minimize topological dependencies between fragments. We utilize the zero-sum and property of the µ2 elements. We split vertices 1 to *n* at *k* into two disjointed sets {*i_1_*,…,*i_k_*} and {*i_k+1_*,…,*i_n_*} by µ2′s sorted elements {ν*_i1_*, ν*_i2_*, …,ν*_ik_*,…, ν*_in_*} where ν*_i1_*≤ν*_i2_*…≤ν*_ik_* ≤…≤ν*_in_* and *k* is determined by a splitting value *s* such that ν*_ik_*≤*s*. We use three standard choices for the splitting value *s* to define three partitioning algorithms: median, sign, and gap. For the median and sign cuts, we select the splitting value *s* as the median of eigenvector elements or as 0, respectively, where in the latter negative values are separated from positives. For the gap cut, the splitting value *s* is in the largest gap in the sorted list of µ2 components. We apply these three methods to all existing graph topologies discovered experimentally up through 11 vertices. Our analysis of the 45 RNA graphs from 4 to 11 vertices shows that the gap cut partitions structures into the most topologically distinct pieces. All resulting subgraphs correspond to existing motifs. Thus, the gap cut appears the most natural for RNA substructuring. Our iterative gap partitioning approach further suggests a systematic procedure to divide a large RNA structure into small RNA motifs and assemble the resulting modules to large RNAs. Permutations of sequences corresponding to the building blocks in the desired order could be used to suggest candidate sequences corresponding to target motifs.

This paper is organized as follows. We begin by describing methods including mathematical formulation of RNA graphs, Laplacian matrices and spectrum, three graph partition algorithms (median, sign and gap cuts) based on the second Laplacian eigenvector µ2, and the RNA data set that we use. We then present results for the topological aspects described by the second Laplacian eigenvector, partitioning results for RNA graphs, and iterative partitioning results. The final discussion provides future directions of the work.

## Methods

### RNA graphs, Laplacian eigenvalues, and eigenvectors

We represent RNA as a graph *G* using RAG tree representation (see Figure 1A). In the RAG tree graphs, RNA 2D structural elements – stems, loops, bulges, and junctions – are converted into 2D graphical objects with the following rules:

1. an edge (−) represents a double-stranded helical stem with more than one base pair.
2. a vertex (•) represents a single strand that occurs in segments connecting 2D structural elements such as bulges, loops, and junctions. Here, a bulge motif is considered to be an internal symmetric or asymmetric loop with more than one unmatched nucleotide or one unstable base pair.

Figure 1 shows three examples – a linear structure 6_1, a 3-way branched structure 6_2, and a 4-way structure 6_5. The RAG graph index shows the total number of vertices and the topological complexity: RAG graph 6_1, 6_2, and 6_5 have six vertices and the subscribed numbers (1, 2, and 5) shows the increased complexity. We label the graph vertices by the order of vertices corresponding to sequences from a 5′ end to a 3′ end.

For a graph *G* = (*V*,*E*) where *V* is the set of labeled vertices and *E* is the set of edges, we define the associated Laplace matrix (called the Laplacian) *L* = (*m_ij_*) = *A
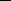
D*, where *A* is an adjacency matrix and *D* is a diagonal matrix, as the *n*×*n* matrix where
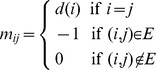
and the value *d(i)* is the total number of edges from vertex *i* and *n* is the total number of vertices (*n* = |*V*|). See Figure 1A for the Laplacian matrices for three graph examples.

Since the Laplacian matrix *L* is symmetric, the eigenvalues of the Laplacian matrix (λ1, λ2, …λn) are non-negative with the smallest eigenvalue λ1 = 0. In the field of the spectral graph theory, the Laplacian eigenvalues and eigenvectors have been extensively studied since they describe combinatorial or topological properties of a graph. In particular, the second smallest eigenvalue, λ2, and its corresponding eigenvector, µ2, provide information about topological compactness and the partitioning properties of graphs, respectively. The second eigenvalue λ2 is positive for any connected graph and increases with the compactness of a graph (see Figure 1A). The second eigenvector µ2 = 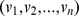 corresponding to λ2 provides local information on the connectivity of each vertex; it also provides information on how to subdivide a large RNA into smaller fragments that minimize dependencies between fragments (see below for details).

Note that the sum of elements of µ2 is 0 (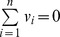) because vector µ1 = (1,1,…,1) is an eigenvector of the Laplacian corresponding to the first eigenvalue (λ1 = 0) and the eigenvectors of a symmetric matrix are orthogonal, i.e., the inner product of (1,1,…,1) and 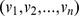 is zero.

### Graph partitioning algorithm

The basic idea in graph partitioning is to split the vertex set *V* into two disjointed sets, A = {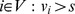} and 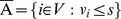, for some given value *s*. Based on the zero-sum property of the second Laplacian eigenvector µ2, a spectral partitioning technique can be used to split the graph according to a splitting value *s* on the basis of the second eigenvector µ2, as elaborated by Spiegelman and Teng on the spectral partitioning technique [38]. We use three standard choices for the splitting value *s*: median, sign, and gap cuts. For the median cut, we select *s* as the median of 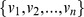, namely *m*, and thus the two sets are {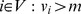} and {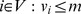}. For the sign cut, we select *s* = 0 and divide *V* into {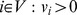} and {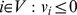}. For the gap cut, we list the eigenvector components 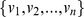 in ascending order 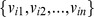, where 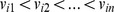; we then calculate the difference of every two neighboring elements (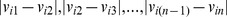), called the “gap”, and select *s* to be the value defining the largest gap: If 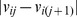 is the largest gap, then 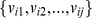 and 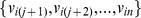 are two disjointed set of vertices by the gap cut. Furthermore, we apply the graph-partitioning algorithm iteratively until all substructured graphs correspond to existing RNAs (the simplest motifs have all structures experimentally deduced). After one partition, a graph *G* becomes two subgraphs, namely, *G_1_* and *G_2_* after one partition. After *k* iterations, *G* becomes *G_1_*, *G_2_*,…, *G_2_^k^* until all *G_i_* correspond to some existing RNA graphs.

### RNA data set

**Figure 2:**
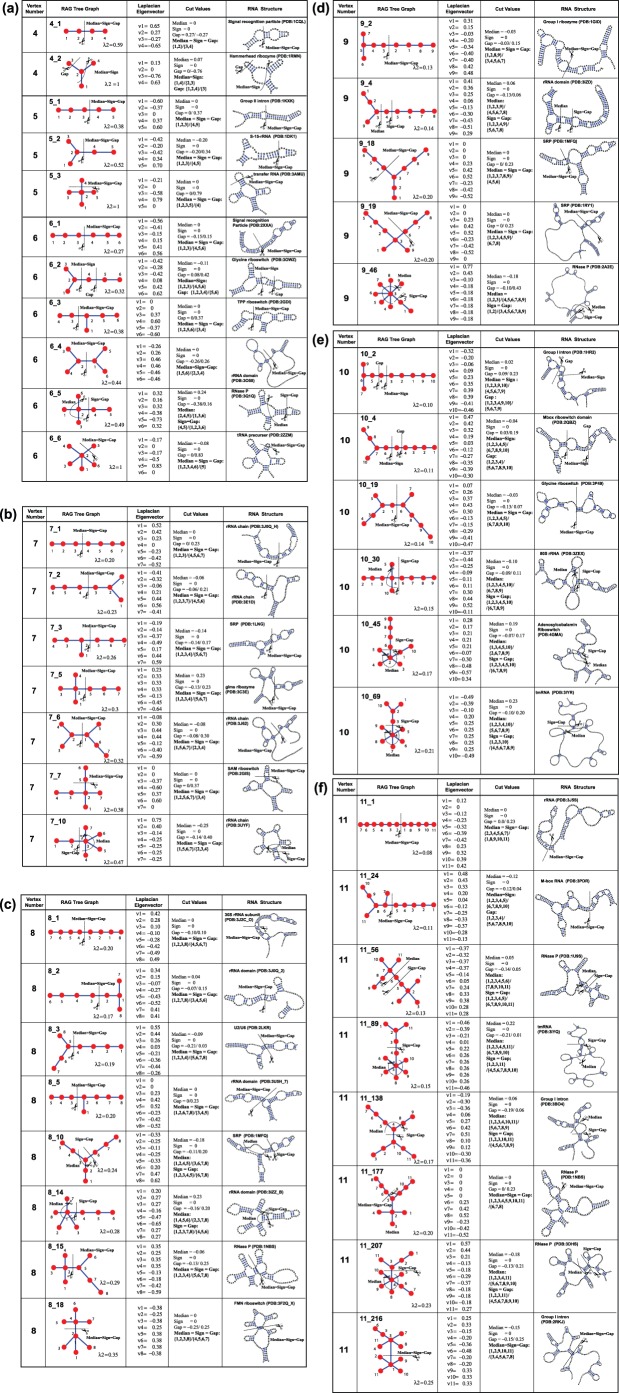
Graph partitioning results for RNAs corresponding to RAG tree graphs with vertex number (a) V = 4, 5, or 6, (b) V = 7, (c) V = 8, (d) V = 9, (e) V = 10, and (f) V = 11. RAG tree graph, Laplacian eigenvector, cut values/results of median/sign/gap partitions, and corresponding RNA secondary structures are shown.

We apply the graph partitioning algorithms to 45 RAG tree graphs with 4 to 11 vertices that have corresponding known structures in the Protein Data Bank (PDB) database (http://www.biomath.nyu.edu/rna, N. Baba et al. in preparation). Note that a graph topology can correspond to multiple RNAs. See Figure 2 for the list of RAG tree graphs and their corresponding secondary structures and partition results.

## Results

### Topological aspects described by the second Laplacian eigenvector

Before describing results of our RNA partitioning, it is useful to understand the overall topologies of RNA graphs. The second eigenvalue (λ2) and eigenvector (µ2) of the Laplacian matrix constructed from the adjacency and degree matrices of each graph describe overall graph connectivity. The eigenvalue λ2 measures the graph complexity. For example, Figure 1A shows that a linear RNA molecule has a smaller λ2 value than a branched molecule: a linear graph 6_1 has λ2 = 0.27, while branched graphs 6_2 and 6_5 have λ2 = 0.32 and λ2 = 0.49, respectively.

The corresponding eigenvector µ2 describes the local topological aspect of a graph. Thus, symmetry produces same numerical components. For example, Figure 1A shows that for Graph 6_1, a simple symmetric linear structure with 6 vertices, µ2 elements increase from 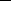
0.56 to 0.56 and have symmetry around 0 ({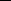
0.56, 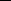
0.41, 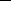
0.15, 0.15, 0.41, 0.56}). For Graph 6_2 with a 3-way branch connected with a linear structure, µ2 is {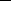
0.42, 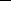
0.28, 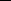
0.42, 0.08, 0.42, 0.62}. For the 3-way structure, the elements are {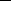
0.42, 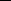
0.28, 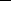
0.42, 0.08}, where the center of the branch corresponds to 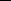
0.28, and the symmetric branches correspond to 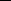
0.42. The last branch connected to a linear structure is 0.08 with two more vertices with increased elements of 0.42 and 0.62. Graph 6_5, a 4-way branch connected with an elongated edge, has µ2 = {0.32, 0.16, 0.32, 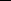
0.38, 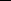
0.73, 0.32}. The vertices 1, 3, and 6, which correspond to the 4-way branch, have the same element value as 0.32. The central vertex 2 has the value 0.16 and the linearly connected vertices have decreasing negative values 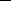
0.38 and 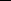
0.73. Since local topological features are reflected by the components of µ2, the ordering of elements can be used to partition graphs.

### Topological aspects of subgraphs partitioned by the median, sign, and gap cuts

From Figure 1B, we see that the partitioning results for the three methods are overall similar for three sample graphs but differ in fine details. Figure 1B shows three examples of graphs – RAG graphs 6_1, 6_2, and 6_5 with partitioning by the median, sign, and gap cuts. For these three graphs, the median cut partitions graphs into two subgraphs with the same number of vertices; the gap cut partitions the graphs into two topologically distinct graphs; and the sign cut results are same as either the median cut or the gap cut, depending on the graph topology. For the linear structure 6_1, all three partitioning have the same result: the linear graph 6_1 is divided into two linear structures with the same vertex numbers ({1,2,3}/{4,5,6}). For the 3-way branched graph 6_2, the median and sign cuts have the same result, with two vertex sets with the same number of three vertices ({1,2,3}/{4,5,6}). The gap cut, however, produces the graph into two distinct topological features: a 3-way branch with four vertices ({1,2,3,4}) and a linear structure with two vertices ({5,6}). For a 4-way branch graph 6_5, the median method partitions the graph into two three-vertex graphs while the sign and gap methods cut the graph into a 4-way branch ({1,2,3,6}) and a linear structure with two vertices ({4,5}).

### Gap cut: best partitioning algorithm for RNA graphs

These three partitioning methods applied to 45 RAG graphs from 4 through 11 vertices corresponding to existing RNAs (whose experimental structures have been deposited in the PDB database) produce the results shown in Figure 2. Here, RAG tree graphs, µ2, the cut values, and results for the small (4–6 vertices, Figure 2a), medium (7–8 vertices, Figures 2b, 2c), and large (9–11 vertices, Figures 2d–f) graphs are shown. The overall cut patterns are similar as analyzed for the simple examples above. Namely, the median cut partitions the graph into two graphs with the same number of vertices; the gap cut partitions the graph into two topologically distinct graphs regardless of the vertex numbers of the partitioned graphs; and the sign cuts coincide with either the median or gap cuts but usually the gap cut. As a result, among 45 existing RNA graphs, 28 cases yield the same results for all three methods. For 13 cases (e.g., graphs 6_5, 8_10, 8_14, 9_4, 10_30, 11_89), the sign and gap cuts produce the same partitioning of the graphs as the high-branched graph plus the linear graphs, while the median cut breaks the branched graphs corresponding to junctions. For four cases, graphs 4_2, 6_2, 10_2, and 10_4, the gap cut keeps the 3-way junction structure and cuts the connected hairpin or internal loop structures. On the other hand, the sign and median cuts have the same partitioning results: junctions are broken to obtain two graphs with equal vertex numbers. Thus, among the three partitioning methods, the gap cut method is well suited for partitioning RNA graphs into basic modules of internal loops, junctions, and hairpins without breaking them, which is usually energetically favorable.

### Iterative gap cuts to partition large RNA

**Figure 3:**
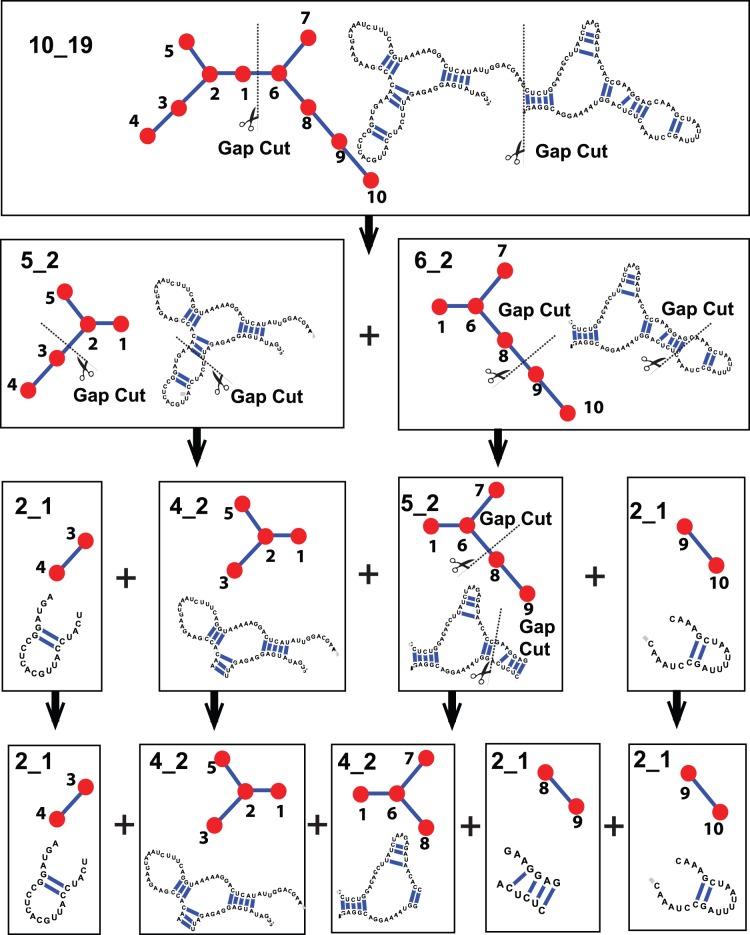
Iterative gap partitioning results for large RNA with example of the glycine riboswitch (PDB code: 3P49, Graph ID: 10_19).

For large RNAs, we apply the gap partitioning iteratively to analyze how basic modules are assembled to make larger ones. For example, we partition graph 10_19 corresponding to the glycine riboswitch (Figure 3), which has two 3-way junctions. After one step of gap partitioning, the two graphs, 5_2 and 6_2, connected by the vertex ID 1 result (for the Laplacian eigenvector elements of RAG ID 10_19, see Figure 2e). The subgraphs 5_2 and 6_2 contain one 3-way junction, and the vertex 1 corresponds to the RNA single strand connecting these two 3-way junctions. After two steps of gap partitioning applied to the second-generation subgraphs 5_2 and 6_2, four third-generation-graphs result: 5_2 becomes 2_1 and 4_2, and 6_2 becomes 5_2 and 2_1. See Figure 2b for the Laplacian eigenvector elements for graphs 5_2 and 6_2. After the third partitioning of 5_2, we produce five minimal modules including two 3-way junctions and three hairpin structures to form the graph 10_19 (Graphs 2_1, 4_2, 4_2, 2_1, and 2_1).

Such an iterative partitioning of gap cuts also suggests hierarchical assembly procedures from the basic modules building up to a large structure by an inverse cutting procedure. For example, to build up an RNA structure of 10_19, we start with five graphs with vertex labels in the fourth row in Figure 3: 2_1 (vertex labels {3,4}), 4_2 (vertex labels {1,2,3,5}), 4_2 (vertex labels {1,6,7,8}), 2_1 (vertex labels {8,9}), and 2_1 (vertex labels {9,10}). The first step of assembly suggested by the iterative gap cut procedure is a combination of graphs 4_2 (vertex labels {1,6,7,8}) and 2_1 (vertex labels {8,9}). From the overlapped vertex labels, the connection point is also suggested: graphs 4_2 (three-way junction) and 2_1 (hairpin loop and dangling end) can be merged through vertex ID 8 to form graph 5_2 (three-way junction and internal loop). The next step suggests assemblies of 2_1 and 4_2 to 5_2 and 5_2 and 2_1 to 6_2, and the final step is assembly of 5_2 and 6_2, to yield the target graph 10_19. This gap cut/assembly procedure indicates that the first step in RNA structure assembly is the connection of junctions to other structures.

## Discussion and Conclusions

We have presented graph-partitioning approaches using three splitting values – median, sign, and gap – based on the Laplacian eigenvector µ2 for understanding modular features of RNA. Our application of these spectral algorithms to all 45 existing RNA graphs from 4 through 11 vertices (http://www.biomath.nyu.edu/rna, N. Baba et al. in preparation) has suggested concrete substructuring as well as design procedures. Namely, sequences corresponding to these basic motifs can be combined in a build-up type optimization we have done for RNAs [21]. Our RAG database has already catalogued both existing RNA topologies and hypothetical RNA graphs, and further classified the latter class into RNA-like or non-RNA-like topologies. For example, there are 42, 100, and 227 graphs with 9, 10, and 11-vertices, respectively, which do not have corresponding existing RNAs but are considered RNA-like (or probable) (N. Baba et al, in preparation). For these RNA-like motifs, a build-up procedure is a reasonable approach.

The RAG tree graph representation of RNA structures captures the global helical connectivity and offers mathematical foundations to measure and analyze RNA topologies. While the second Laplacian eigenvalue λ2 describes the overall compactness of the graph, the elements of second Laplacian eigenvector µ2 describe the topological contributions/locations of each vertex to the overall motif. For example, a linear structure has simply increasing or decreasing µ2 elements depending on how the vertices are labeled (see Graphs 4_1, 5_1, 6_1 in Figure 2a, Graph 7_1 in Figure 2b). In a branched structure, the vertices of branch ends have the same µ2 components (for example, see Vertex ID 1 and 3 of Graph ID 5_2 and 6_2 in Figure 2a).

Thus, partitioning vertices into two groups by the gap cut algorithm provides a mathematically reasonable and biologically relevant graph partitioning result. The median cut partitions a graph into two equal-sized subgraphs. The largest gap cut partitions a graph into two topologically distinct subgraphs. For example, graph 6_2 in Figure 2a has two 3-vertex subgraphs, but the gap cut produces one 4-vertex subgraph with a junction structure and another 2-vertex subgraph plus a linear structure.

Since junction structures cannot easily be divided energetically, the gap cut suggests a quantitative and systematic approach to describe basic modules and their hierarchical assembly. As shown in the example of the graph of 10_19 in Figure 3, our algorithm suggests how a large RNA can be built from RNA building blocks. The gap partitioning can be utilized to design RNA sequences that fold into the target graph, which could help to expand the structural repertoire of RNAs.

Our prior design work by build-up has used simple division of ten dual graphs to predict novel motifs [21]. Already half of them were experimentally determined by different methods such as X-ray crystallography, nuclear magnetic resonance spectroscopy, or comparative analysis of genomic sequences [20]. We further observed high sequence similarity between designed and actual RNA sequences, much greater than expected by chance (25%) [20]. However, such an ad-hoc build-up procedure cannot be used to design complex pseudoknot structures systematically [20].

The application of our partitioning approach for RNA tree graphs to RNA design is limited to pseudoknot-free structures. Since pseudoknot motifs are important for general RNA applications, build-up approaches based on partitioning algorithms for dual graphs are required. In particular, dealing with self-loops (edges connecting the same vertex) requires further development. It may also be possible to approach pseudoknot partitioning using modified tree graphs that have additional edges or graph elements accounting for pseudoknot interactions.

Our work here is the first application of standard graph partitioning algorithms to the area of biology. The partitioning procedures can be extended to the design of novel RNA motifs by assembling modules corresponding to the subgraphs identified from partitioning, as we have done previously for ten specific motifs [21]. Of course, topology is just one aspect. Chemistry and thermodynamics must be considered as well. For example, free energy calculations and energy landscape analyses are further needed to screen in-silico designed sequences that fold onto the target topologies. In our analysis of RNA riboswitch energy landscapes, for example, we found the distribution and barrier type of the conformational clusters in riboswitch energy landscapes to be useful for discriminating various riboswich classes depending on the thermodynamic control of ligand-binding [31], [32].

A generalization of RNA graph partitioning to other areas of biology can certainly be envisioned. Many biological networks, such as metabolic pathways associated with biochemical reactions and regulatory protein interaction networks, have been constructed by organizing building blocks. Thus, the application of graph partitioning to these systems could define a valuable tool to identify the modularity of many networks in biology, engineering, and medicine.
